# MiR-23a induced the activation of CDC42/PAK1 pathway and cell cycle arrest in human cov434 cells by targeting FGD4

**DOI:** 10.1186/s13048-020-00686-9

**Published:** 2020-08-09

**Authors:** Ji Lin, Huijuan Huang, Liheng Lin, Weiwei Li, Jianfen Huang

**Affiliations:** 1 Graduate School, Fujian Medical University, Fuzhou, China; 2 The 900th hospital of the Joint Service Support Force of the Chinese People’s Liberation Army, Fuzhou, China; 3 Gynaecology, Mindong Hospital in Ningde City, No. 89 Heshan Road, Fuan, Fujian China

**Keywords:** miR-23a, Polycystic ovary syndrome, FGD4, Binding site, Cell cycle

## Abstract

**Background:**

MiRNAs play important roles in the development of ovarian cancer, activation of primitive follicles, follicular development, oocyte maturation and ovulation. In the present study, we investigated the specific role of miR-23a in cov434 cells.

**Results:**

Downregulation of miR-23a was observed in serum of PCOS patients compared with the healthy control, suggesting the inhibitory effect of miR-23a in PCOS. MiR-23a was positively correlated with Body Mass Index (BMI) and negatively correlated with Luteinizing hormone (LH), Testostrone (T), Glucose (Glu) and Insulin (INS) of PCOS patients. MiR-23a mimic inhibited the proliferation and promoted apoptosis of human cov434 cells. In addition, flow cytometry assay confirmed that miR-23a blocked cell cycle on G0/G1 phase. MiR-23a inhibitor showed opposite results. Furthermore, double luciferase reporter assay proved that miR-23a could bind to the 3’UTR of FGD4 directly through sites predicted on Target Scan. FGD4 level was significantly suppressed by miR-23a mimic, but was significantly enhanced by miR-23a inhibitor. We further proved that miR-23a increased the expression of activated CDC42 (GTP bround) and p-PAK-1, suggesting that miR-23a induced cell cycle arrest through CDC42/PAK1 pathway.

**Conclusions:**

In conclusion, our study reveals that miR-23a participates in the regulation of proliferation and apoptosis of cov434 cells through target FGD4, and may play a role in the pathophysiology of PCOS.

## Background

Polycystic ovary syndrome (PCOS) is the most common reproductive, endocrine and metabolic disorder disease in women, characterized by ovulation disorders, hyperandrogenism and insulin resistance [1, 2]. PCOS affects about 5–10% of women of childbearing age, accounting for 75% of anovulatory infertility, and usually a lifelong disease. Its common clinical manifestations include menstrual disorders, sub-fertility, acne vulgaris, alopecia, seborrheia, obesity, hirsutism and acanthosis [3]. Women with PCOS have an increased risk of insulin resistance, hypertension, type 2 diabetes, oxidative stress, dyslipidemia, cardiovascular disease and endometrial cancer [4]. Therefore, understanding the molecular mechanism of metabolic diseases underlying the pathophysiology of PCOS will help to identify new diagnostic and therapeutic strategies. In addition, although the exact etiology of PCOS remains to be understood, it has been clear that the survival and proliferation of granulosa cells are closely related to the pathogenesis of PCOS [5].

In recent years, the role of microRNAs (miRNAs) in ovarian physiology and pathology has attracted much attention. Some studies have shown that miRNAs play important roles in the development of ovarian cancer, activation of primitive follicles, follicular development, oocyte maturation and ovulation [6–8]. Several studies have found a variety of differentially expressed microRNAs in ovarian granulosa cells of PCOS patients, which are closely related to the proliferation and apoptosis of ovarian granulosa cells, and the production of progesterone, estradiol and testosterone [9, 10].

The human miR-23a gene is located on chromosome 19 of the human genome and transcribed into a part of the miR-23a-27a-24-2 cluster [11]. Mi-23a-27a-24-2 cluster, which encodes primicroRNA transcripts composed of three kinds of miRNAs (miR-23a, miR-27a and miR-24-2), is responsible for inducing caspase-dependent and caspase-independent apoptosis of embryonic kidney cells (HEK293T) through human c-Jun N-terminal kinase pathway [11]. In recent years, more and more evidence has shown that miR-23a is essential for folliculogenesis. It has been reported that the expression of circulating miR-23a of patients with PCOS was down-regulated compared with healthy women, and proved that miR-23a is a better indicator for evaluation of PCOS than the miR-23b [12]. However, as far as we know, the specific role and mechanism of miR-23a in PCOS have not been studied.

Studies proved that miR-23a is significantly up-regulated in premature ovarian insufficiency (POI) patients’ serum and poor ovarian response (POR) patients’ ovarian granulosa cells [13–16]. Compared with normal women, miR-23a was significantly upregulated in follicular cells of women receiving assisted reproductive technology (ART) due to oviduct and endometriosis [17]. More critically, miR-23a can promote the apoptosis by affecting the expression of multiple targets, including XIAP, SMAD5 and Sirt1 [14, 15, 18].

Therefore, in the present research, we hypothesized that miR-23a is involved in the development of PCOS by regulating downstream pathways related to cell survival in ovarian cells. The objective of this study was to confirm the regulatory effect and mechanism of miR-23a on the growth of cov434 cells. We analyzed the expression of miR-23a in serum samples from PCOS patients and healthy women and the correlation between miR-23a level and PCOS symptoms. We focused on a new molecular mechanism by which miR-23a induces apoptosis in granular cells.

## Materials and methods

### Samples

The serum of 50 Chinese women with PCOS was collected in Mindong hospital, Ningde City, Fujian Province from September 2018 to December 2018. According to the revised PCOS diagnostic criteria published by the Rotterdam consensus [1], the PCOS group excluded patients with Cushing’s syndrome, delayed congenital adrenal hyperplasia, thyroid dysfunction / hyperthyroidism, hyperprolactinemia or androgen secreting tumor, as well as patients with diabetes, hypertension, chronic kidney disease, smoking and using alcohol or drugs. The serum of 50 healthy women was collected as the control group. The volunteers in the control group had normal menstruation, normal ovaries and no history of reproductive system disease or appendicitis. The control and PCOS group did not take any medications in the past 3 months, including oral contraceptives or other hormonal medications with no intrauterine devices or smoking. Patients with reproductive system disease or appendicitis history were excluded from the control group. All volunteers had understood the purpose and requirements of this study and signed a written informed consent before participating in the study. 4 ml of elbow venous blood from each sample was taken and stored in a refrigerator at − 80 °C. All the experiments involved in this study have obtained the ethical approval of Mindong hospital in Ningde City.

### Evaluation of BMI and sex hormone

The weight and height of the volunteers were measured to calculate Body mass index (BMI) (BMI = weight/height^2^). Radioimmunoassay (RigorBio Scientific and Technology Co., Beijing) was used to measure the level of total testosterone and other sex hormones.

### Cell line and transfection

Cell lines KGN (derived from a granulosa cell tumor), cov434 (derived from a granulosa cell tumor) and SVOG (derived by immortalization of granulosa/luteal cells using SV40 large T antigen) were purchased from cell resource bank of Chinese Academy of Sciences. 1 × 10^5^ cells were seeded into 24 well plates. MiR-23a micmic (50 nM), miR-23a inhibitor (100 nM) and negative control (NC, 50 nM mimic NC and 100 nM inhibitor NC) (Ruibo Biotechnology Co., Ltd., Guangzhou, China) were transfected into cov434 cells by Lipofectamine™ 2000. Normal untreated cov434 cells were cultured as control. The sequence of siRNA used in this study is as follows: miR-23a mimic, 5′-CCTTTAGGGACCGTTACACTA-3′; mimic NC, 5′-TTCTCCGAACGTGTCACGTTTC-3′; miR-23a inhibitor, 5′-TAGTGTAACGGTCCCTAAAGG-3′; inhibitor NC, 5′-TTCTCCGAACGTGTCACGTTTC-3′.

### Real time fluorescence quantitative PCR (qPCR)

Total RNA were extract from samples or cells using Trizol reagent. Related expression of target gene was calculated using 2^-ΔΔCt^ method. This study involves the following sequences: miR-23a-3p Reverse transcription: 5′- GTCGTATCCAGTGCAGGGTCCGAGGTATTCGCACTGGATACGACGGAAAT-3′; miR-24-2 Reverse transcription: 5′-GTCGTATCCAGTGCAGGGTCCGAGGTATTCGCACTGGATACGACCTGTGT-3′; miR-27a-3P 5′-GTCGTATCCAGTGCAGGGTCCGAGGTATTCGCACTGGATACGACGCGGAA-3′; U6 Reverse transcription, 5′-AAAATATGGAACGCTTCACGAATTTG-3′; miR-23a-3p forward primer 5′-GCGATCACATTGCCAGGG-3′ and reverse primer 5′-AGTGCAGGGTCCGAGGTATT-3′; miR-24-2 forward primer 5′-CGCGTGCCTACTGAGCTGAA-3′ and reverse primer 5′-AGTGCAGGGTCCGAGGTATT-3′; miR-27a-3P forward primer 5′-GCGCGTTCACAGTGGCTAAG-3′ and reverse primer 5′-AGTGCAGGGTCCGAGGTATT-3′; U6 forward primer 5′-CTCGCTTCGGCAGCACATATACT-3′ and reverse primer 5′-ACGCTTCACGAATTTGCGTGTC-3′; FGD4 forward primer 5′-CCTGCCTCTGCTTCTTGTGTCTC-3′ and reverse primer 5′-TGGTTGTCAATCCATGCCTTCCTG-3′.

### Cell proliferation assay

After 12 h of transfection, cells were seeded into a 96 well plate at the density of 5 × 10^3^ cells per well. Each group of cells was treated with 6 replicates. After incubation for the specified time (0, 12, 24, 48 and 72 h), 10 μl of CCK-8 reagent was added and incubated at 37 °C for 2 h. The absorbance of each pore was measured at 450 nm by an enzyme labeling instrument.

### Flow cytometry analysis for cell cycle

After 48 h of transfection, the cell cycle was detected by flow cytometry. The cells were fixed with 70% ethanol overnight at 4 °C. The cells were resuspended with 500 μl of binding buffer. 50 μl PI was added to the cell suspension and incubated at room temperature for 30 min. The results were analyzed by ModFit and displayed by FL2-w and FL2-a.

### Flow cytometry analysis for apoptosis

After 48 h of transfection, the apoptotic cells were detected by flow cytometry. 2 μl of PI and FITC annein V were added into 100 μl cell suspension and incubated at room temperature for 10 min. Cell apoptosis was detected using a flow cytometer.

### Western blot

The total protein was extracted with RIPA buffer. BCA method was used to detect the protein concentration. The extracted protein was electrophoresis by SDS-PAGE and transferred to PVDF membrane. PVDF membrane was incubated in 5% skimmed milk at room temperature for 1 h, and then primary antibody overnight at 4 °Cfollowed by the secondary antibody at room temperature for 2 h. QUANTITY ONE software is used for result analysis. The following antibodies were used in this research: anti-FGD4 (Abcam, ab97785, 1:2000, 87KDa); anti-CDC42 (Abcam, ab155940, 1:1000, 21KDa); anti-PAK1 (Abcam, ab223849, 1:1000, 60KDa) and β-actin (TransGen Biotech, HC201, 1:5000, 42KDa).

### Double luciferase reporting assay

The plasmids of wild type (FDG4-WT) and mutant type (FDG4-MUT) luciferase reporter genes were constructed using pcDNA3.1as the empty vector. MiR-23a mimic, mimic NC, FDG4-WT and FDG4-MUT plasmids were co-transfected into cov434 cells by LipofectamineTM 2000. Cells were divided into four groups: FGD4-WT 3′-UTR + miR-23a mimic NC; FGD4-Mut 3′-UTR + miR-23a mimic NC; FGD4-WT 3′-UTR+ miR-23a mimic; FGD4-Mut 3′-UTR+ miR-23a mimic. After 36 h of transfection, Firely Lueiferase (F) and Renilla Luciferase (R) were detected by GLO-MAX 20/20 \fluorescence detector, and the relative luciferase activity (F / R) was calculated.

### Statistical analyses

All data were analyzed with SPSS 22.0 (SPSS, Inc., Chicago, IL) software, and represented as mean ± SD. Spearman method was used to analyze the relationship between miRNA level and other indicators. Independent sample t-test was used to evaluate the difference between two groups and One-way ANOVA was used to analyze the difference between three and more groups with post hoc contrasts by Bonferroni test. *P* <  0.05 was considered statistically significant.

## Results

### MiR-23a was downregulated in serum of PCOS patients

**Fig. 1:**
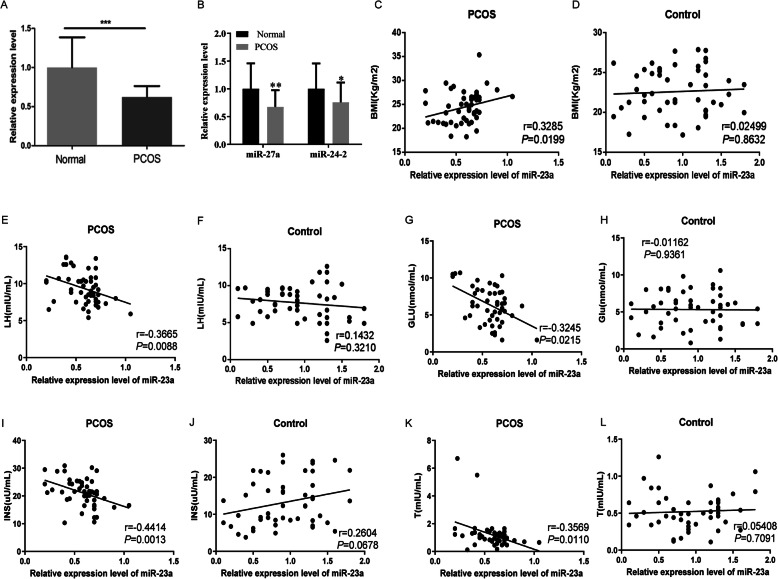
MiR-23a was downregulated in serum of PCOS patients. **a** qPCR was performed to detect the expression of miR-23a in PCOS sample (PCOS) and healthy control (Normal). **b** miR-27a and miR-24-2 levels were detected using qPCR in PCOS and normal group. Correlation between miR-23a level and BMI was analyzed in PCOS (**c**) and control (**d**) group. Correlation between miR-23a level and LH was analyzed in PCOS (**e**) control (**f**) group. Correlation between miR-23a and GLU level was analyzed in PCOS (**g**) and control (**h**) group. Correlation between miR-23a and INS level was analyzed in PCOS (**i**) control (**j**) group. Correlation between miR-23a and T level was analyzed in PCOS (**k**) and control (**l**) group. *P < 0.05; **P < 0.01; ***P < 0.001

Peripheral blood was collected from 50 local PCOS patients for the detection of miR-23a level with 50 healthy women’s peripheral blood as the control. Clinical information on age, BMI and sex hormone levels of PCOS patients and normal control samples are all listed in Table 1. As shown in Fig. 1a, the serum miR-23a level in PCOS patients was significantly lower than that in the control group (*P* <  0.001). Then, we detected the level of miR-27a and miR-24-2 using qPCR. As shown in Fig. 1a, miR-27a and miR-24-2 also downregulated in peripheral blood of PCOS patients compared with healthy sample.

**Table 1:** The clinical information of PCOS and control groups

Clinical index	PCOS (*n* = 50)	Control (*n* = 50)	*P*
Age	30.98 ± 3.82	30.76 ± 3.12	0.753
E2 (pg/mL)	41.34 ± 12.05	37.65 ± 11.32	0.118
BMI (Kg/m^2^)	24.48 ± 2.62	22.63 ± 2.48	< 0.001
LH (mIU/mL)	9.35 ± 1.77	7.67 ± 1.80	< 0.001
FSH (mIU/mL)	8.22 ± 1.10	7.79 ± 1.12	0.051
PRL (mIU/L)	270.3 ± 132.5	227.9 ± 116.6	0.091
T (mIU/mL)	1.19 ± 0.53	0.522 ± 0.18	< 0.001
Glu (nmol/mL)	6.33 ± 2.00	5.31 ± 1.89	< 0.05
INS (μU/mL)	21.19 ± 3.79	13.25 ± 5.46	< 0.001

*E2* Estradiol, *BMI* Body Mass Index, *LH* Luteinizing hormone, *FSH* Follicule-stimulating hormone, *PRL* Prolactin, *T* Testostrone, *Glu* Glucose, *INS* Insulin

### The correlation between the expression of miR-23a and clinical index of PCOS patients

We further analyzed the correlation between the expression of miR-23a and clinical index. As shown in Table 1, the BMI of PCOS patients were significantly higher than that of healthy controls (*P* <  0.001). The correlation analysis showed that there was a positive correlation between serum miR-23a level and BMI in PCOS patients (Fig. 1b, *P* = 0.0199, *r* = 0.3285), but no correlation was found in healthy control group (Fig. 1c, *P* = 0.8632, *r* = 0.02499). As shown in Table 1, the serum LH concentration in PCOS patients was 9.35 ± 1.77 mIU/mL, which was significantly higher than that in healthy women (7.67 ± 1.80 mIU/mL) (*P* <  0.001). Furthermore, there was a negative correlation between serum miR-23a level and LH concentration in PCOS patients (Fig. 1d, *P* = 0.0088, *r* = 0.3665), but no correlation was found in healthy control group (Fig. 1e, *P* = 0.3210, *r* = 0.1432). The serum miR-23a level was also negative correlated with GLU (Fig. 1f, *P* = 0.0215, *r* = 0.3245), INS (Fig. 1h, *P* = 0.0013, *r* = 0.4414) and T (Fig. 1j, *P* = 0.0110, *r* = 0.3569) concentration in PCOS patients, but not in healthy control group (GLU: Fig. 1g, *P* = 0.9361, *r* = 0.0116; INS: Fig. 1i, *P* = 0.0678, *r* = 0.2604; and T, Fig. 1k, *P* = 0.7091, *r* = 0.0541).

### MiR-23a inhibits the proliferation of cov434 cells

**Fig. 2:**
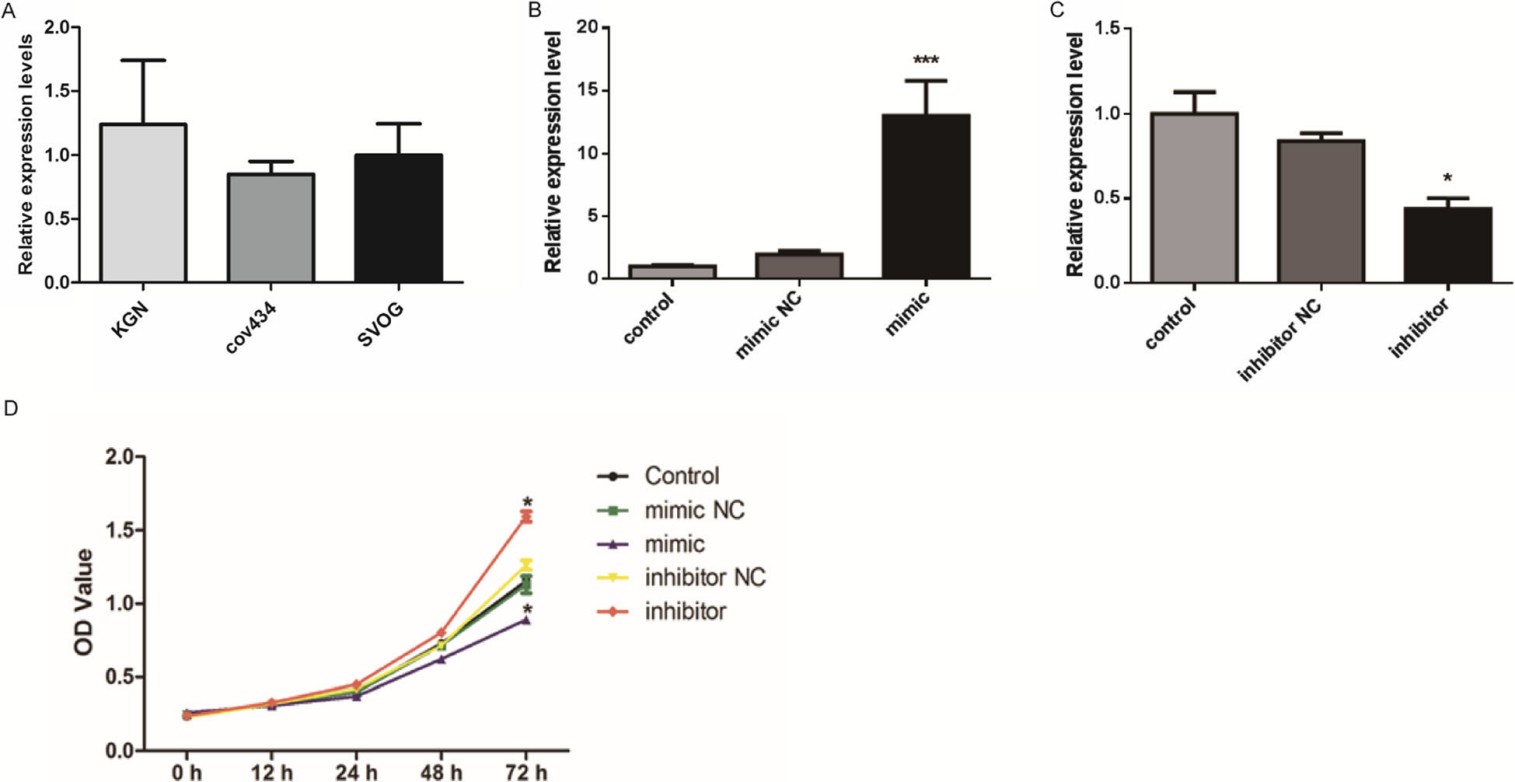
MiR-23a inhibits the proliferation of human ovarian granulosa cells. **a** The expression of miR-23a in three human ovarian granulosa cell lines KGN, cov434 and SVOG was detected by qPCR. **b** MiR-23a was overexpressed by the transfection of miR-23a mimics. **c** MiR-23a was knocked down by the transfection of miR-23a inhibitor. **d** CCK8 was performed to detect the proliferation of cov434 cells. *P < 0.05; ***P < 0.001

In this study, the expression of miR-23a in three human granulosa cell lines was detected by qPCR. As shown in Fig. 2a, the expression level of miR-23a was lowest in cov434 cells and highest in KGN cells. Therefore, we chose cov434 cell line for subsequent experiments. Subsequently, miR-23a-specific-siRNA or mimic was transfected into cov434 cells to explore the role of miR-23a. As shown in Fig. 2b, the expression of miR-23a in cells was significantly increased by the transfection of miR-23a mimic (*P* <  0.001). Similarly, the expression of miR-23a in cells was significantly knocked down by the transfection of miR-23a inhibitor (Fig. 2c) (*P* <  0.05).

Then, CCK8 assay was performed to detect the effect of miR-23a on the proliferation of cov434 cells. As shown in Fig. 2d, compared with the control group, the transfection of miR-23a mimic significantly inhibited the proliferation of cov434 cells (*P* <  0.05); on the contrary, the transfection of miR-23a inhibitor significantly promoted the proliferation of cov434 cells (*P* <  0.05). These data proved that the expression level of miR-23a was involved in the regulation of cov434 cell proliferation.

### MiR-23a induced cell cycle arrest on G0/G1 phase of cov434 cells

**Fig. 3:**
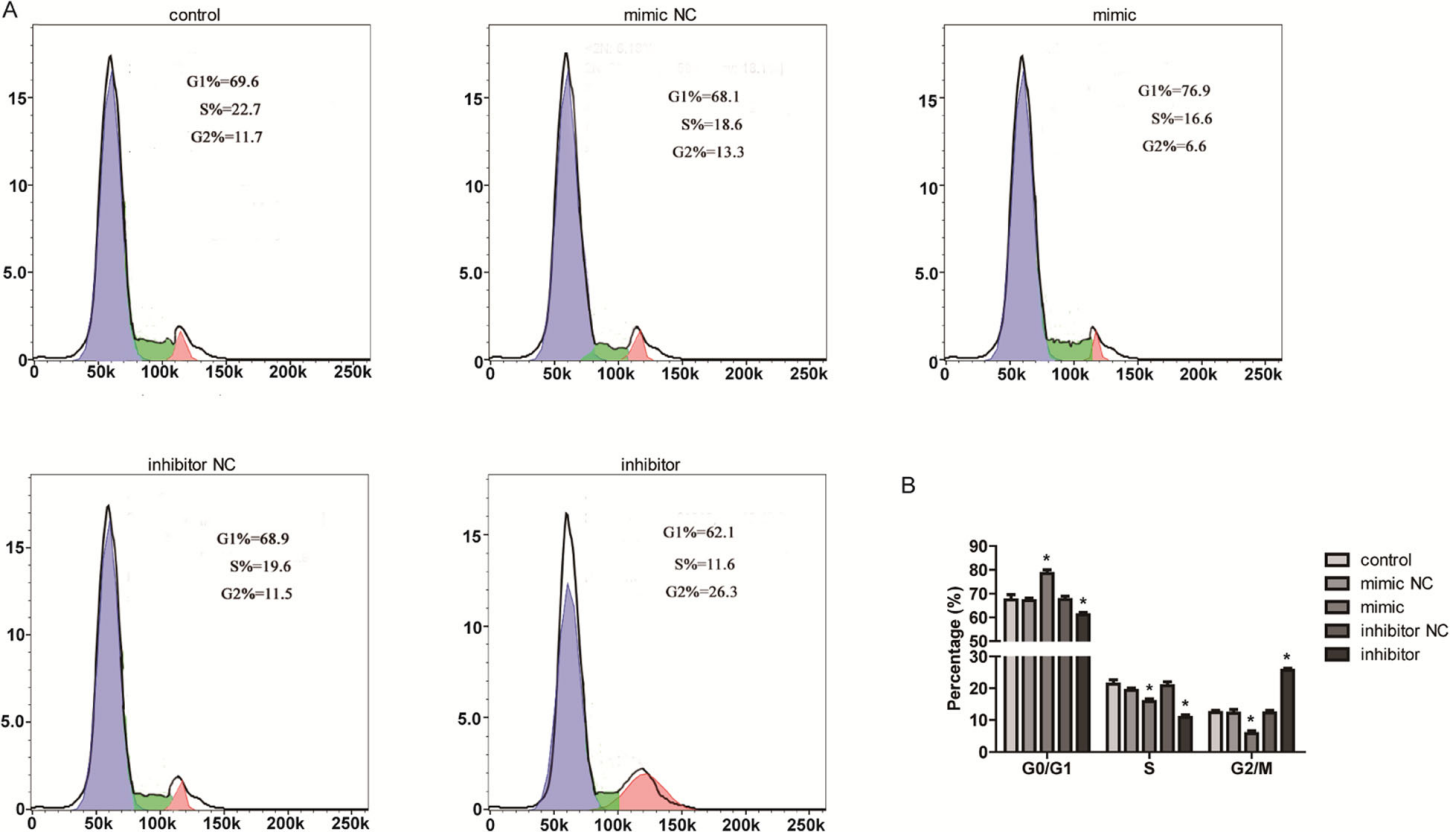
MiR-23a induced cell cycle arrest on G0/G1 phase of cov434 cells. **a** Flow cytometry was used to detect the effect of miR-23a on the cell cycle of cov434 with transfection of miR-23a mimics or inhibitor. **b** Column diagram showed the analysis of cell cycle. *P < 0.05

Next, flow cytometry was used to detect the effect of miR-23a on the cell cycle of cov434. As shown in Fig. 3, cells stagnated in G0/G1 phase after transfection of miR-23a mimic (*P* <  0.05), and the proportion of cells in S phase and G2/M phase decreased significantly (*P* <  0.05). The results were consistent with the inhibition of cell proliferation by over-expression of miR-23a, suggesting that miR-23a induced cell cycle arrest and thus inhibit cell proliferation in cov434 cells. On the contrary, the proportion of G2/M phase cells increased significantly in the miR-23a inhibitor group (*P* < 0.05), while that of G0/G1 and S phase cells decreased (*P* < 0.05). The results showed that low expression of miR-23a promoted cell cycle progression and thus cell proliferation.

### MiR-23a promotes apoptosis of cov434 cells

**Fig. 4:**
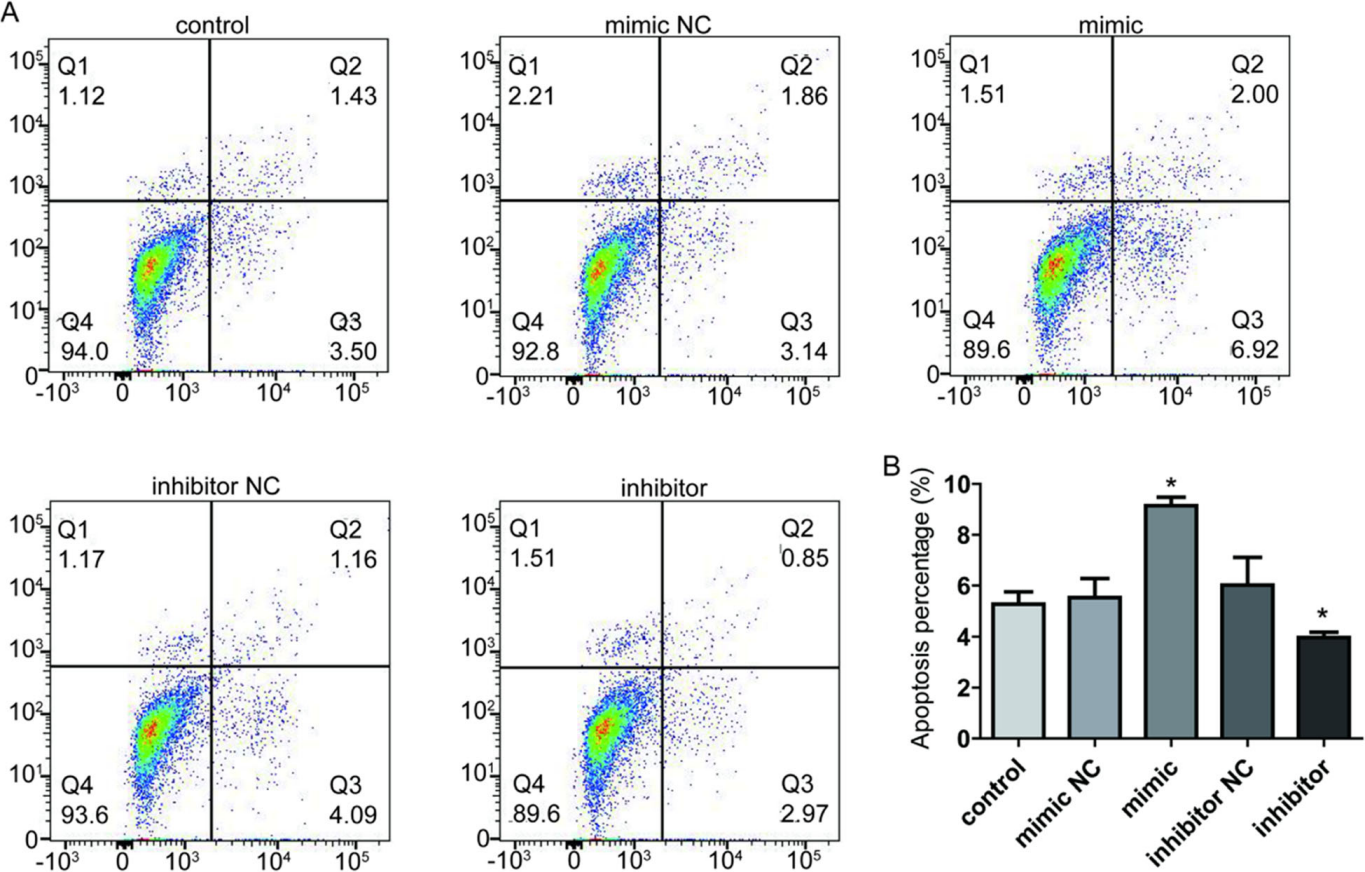
MiR-23a promotes apoptosis of human ovarian granulosa cells. **a** Flow cytometry was used to detect the effect of miR-23a on the apoptosis of cov434 with transfection of miR-23a mimics or inhibitor. **b** Column diagram showed the analysis of cell apoptosis. *P < 0.05

Flow cytometry was performed to detect the effect of the expression of miR-23a on the apoptosis of cov434 cells. As shown in Fig. 4, apoptotic cells increased significantly (*P* < 0.05) after the transfection of miR-23a mimic, and decreased significantly (*P* < 0.05) after the transfection of miR-23a inhibitor. These results suggested that overexpression of miR-23a promoted apoptosis, while low expression of miR-23a inhibited apoptosis.

### FGD4 is the bind target of miR-23a in cov434 cells

**Fig. 5:**
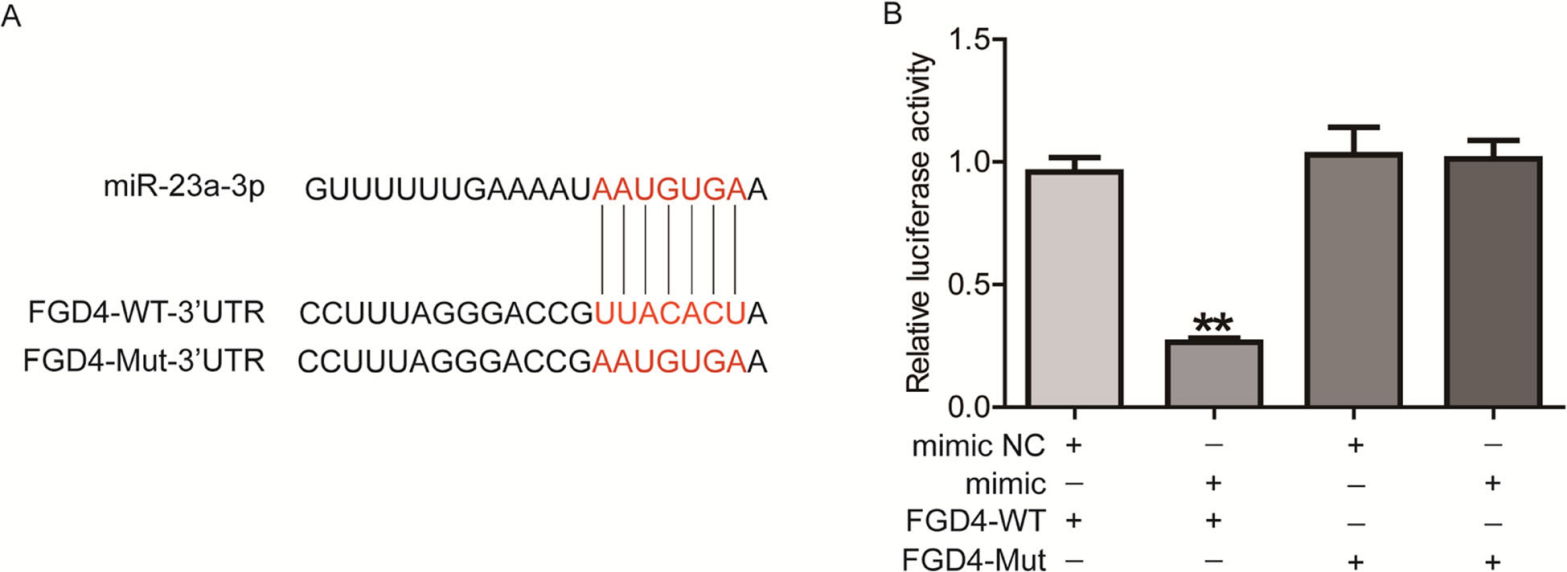
FGD4 binds to miR-23a via the 3’UTR in cov434 cells. **a** the binding site of miR-23a to 3’UTR of FGD4. **b** Double luciferase reporter assay was performed to confirm the binding between miR-23a and FGD4’s 3’UTR. **P < 0.01

Then, we predicted six novel potential target of miR-23a via the analysis on bioinformatics software Target Scan. Subsequently, the results of double luciferase reporter assay proved that only FGD4 could bind to miR-23a directly through predicted sites. The binding sites are shown in Fig. 5a. Co-transfection of miR-23a mimic inhibited the luciferase activity of FGD4-WT plasmid (*P* < 0.01), but had no effect on the luciferase activity of FGD4-Mut plasmid (Fig. 5b). The results showed that miR-23a and FGD4 bind directly through predictive sites.

**Fig. 6:**
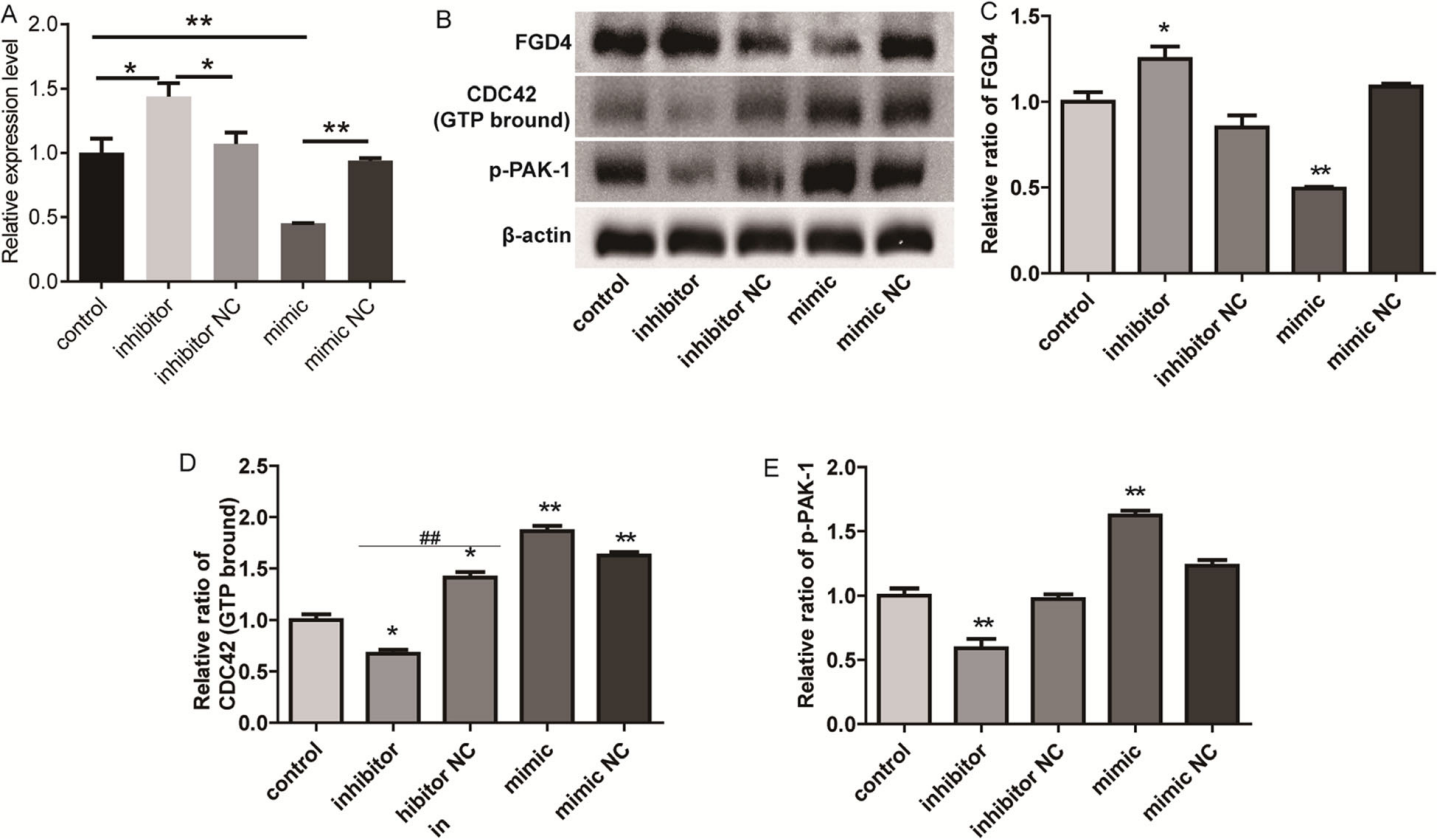
MiR-23a induces the activation of CDC42/PAK-1 signaling pathway in cov434 cells. **a** The expression of FGD4 was detected using qPCR in cov434 cells with transfection of miR-23a mimics or inhibitor. **b** Western blot was performed to detect the levels of CDC42 and p-PAK-1 in cov434 cells with transfection of miR-23a mimics or inhibitor. **c** Column diagram showed the expression level of FGD4. **d** Column diagram showed the expression level of CDC42. **e** Column diagram showed the expression level of p-PAK-1

The effect of miR-23a on the expression of FGD4 in cov434 cells was investigated using qPCR and western blot. As shown in Fig. 6a, the expression of FGD4 was significantly decreased by the transfection of miR-23a mimic (*P* < 0.01), whereas the transfection of miR-23a inhibitor significantly increased the mRNA expression of FGD4 in cov434 cells (*P* < 0.05). As shown in Fig. 6b and c, the protein level of FGD4 was significantly decreased by the transfection of miR-23a mimic (*P* < 0.01), whereas the protein level of FGD4 was significantly increased by miR-23a inhibitor (*P* < 0.05). Combining with the double Luciferase Report experiment, these results indicated that miR-23a physically bind to the 3′-UTR region of FGD4, thereby regulating the level of FGD4 in cov434 cells.

### MiR-23a induces the activation of CDC42/PAK-1 signaling pathway in cov434 cells

CDC42 is a member of the Rho GTPase protein family. FGD4 is responsible for activating CDC42 through GTP exchange of GDP. PAK-1, a serine/threonine kinase, was initially identified as a protein interacting with CDC42 [19]. CDC42/PAK-1 signaling pathway involved in the regulation of cell proliferation, apoptosis and cell cycle [19]. As shown in Fig. 6d, the protein expression of activated CDC42 (GTP bround) was significantly increased by the transfection of miR-23a mimic (*P* < 0.01), and significantly decreased by the transfection of miR-23a inhibitor (*P* < 0.05). The effect of miR-23a on the expression of p-PAK-1 protein was similar to that of CDC42 protein (Fig. 6f).

## Discussion

In this study, we explored the differences in serum levels of miR-23a between PCOS patients and normal women, as well as the effects of miR-23a on biological behavior such as proliferation and apoptosis of cov434 cells and related specific molecular mechanisms, in order to provide limited theoretical support and experimental data for the application of miRNA in PCOS treatment.

Firstly, we found that compared with healthy women, the serum level of miR-23a in PCOS patients decreased significantly. According to previous reports, the level of miR-23a in patients with ovarian disease remains uncertain. Yang et al. reported that miR-23a was highly expressed in the plasma from 39 premature ovarian failure (POF) patients compared with 20 controls with a fold change 2.75 [14]. However, Dang et al. found that miR-23a is down-regulated in the plasma of Chinese patients with premature ovarian failure [15]. This inconsistency may be caused by individual differences and low sample size. MiR-23a level in patients with ovarian disease still needs to be verified in a large number of samples.

Moreover, miR-23a was positively correlated with BMI and negatively correlated with serum LH, T, Glu and INS concentration. Hyperandrogenism and hyperinsulinemia in PCOS patients are the most important physiological changes, exacerbating endocrine disorders [20]. MiR-23a is closely related to the changes of hormone levels, suggesting that it may be involved in the progression of PCOS and is a potential clinical treatment target. Murri et al. also reported an inverse relationship between BMI and LH concentrations in patients with PCOS [21]. Serum is composed of multiple components from a variety of tissues and organs. Therefore, the concentration of miR-23a in serum is regulated by a variety of components and factors. In addition, the results also indicated that the decrease in miR-23a had a negative impact on the occurrence of PCOS and the increase in LH.

Then, we investigated the role of miR-23a in cov434 cells. We have found that miR-23a can affect the proliferation of cov434 cells by regulating cell cycle and participate in the regulation of cell apoptosis through a series of cell functional studies. It has been shown that miR-23a is closely related to apoptosis by inhibiting the expression of Apaf-1 and Bcl-2 apoptotic proteins (including Noxa, Puma and Bax) in neurons [22]. It has also been reported that miR-23a protects differentiated embryonic stem cells from apoptosis induced by bone morphogenetic protein 4 (BMP-4) by targeting SMAD5 [23]. These data provide strong support for our results, suggesting that miR-23a may be closely related to granulosa cell apoptosis through a variety of pathways.

These results suggest that miR-23a may be closely related to the pathogenesis and development of PCOS. Therefore, we further study the molecular mechanism of miR-23a involved in the proliferation and apoptosis of cov434 cells. The biological functions of miRNAs depend mainly on their effects on targets. The same microRNAs may have hundreds of target proteins those change with cell type and cell state. MiR-23a can promote the apoptosis of cov434 cells by affecting the expression of multiple targets [14, 18, 23, 24]. At present, many targets have been found including X-linked inhibitor of apoptosis protein (XIAP), SMAD5 and Sirt1 [14, 18]. In this study, we found FGD4 as a new target of miR-23a. The direct interaction between the 3′-UTR region of FGD4 mRNA and the expression of miR-23a was demonstrated by double luciferase reporter assay. The results of qPCR and Western blot showed that over-expression of miR-23a inhibited the expression of FGD4 at the level of protein and mRNA, while low expression of miR-23a promoted the expression of FGD4 at the level of protein and mRNA.

FGD4 is a Guanine Nucleotide Exchange Factor (GEF) specific to CDC42 Rho GTPase and also an F-actin binding protein, which is essential for maintaining myelin formation in Schwann cells [25]. FGD4 consists of N-terminal F-actin binding(FAB) domain, Dbl homology (DH) domain, two pleckstrin homology (PH) domain and FYVE domain [25]. FGD4 has many functions, including binding to F-actin through FAB domain, activating Rho GTPase signal transduction pathway by increasing the concentration of CD42 binding to GTP. The structure domain of FGD4 indicates that it acts as a cross-linker between membrane structure and actin cytoskeleton; therefore, the functional deletion mutation of FGD4 coding gene may result in truncated FGD4 expression and lead to motor sensory neuropathy or Charcot-Marie-Tooth(CMT) type 4 [26, 27]. The mutation is mediated by inhibiting guanine nucleotide exchange, leading to the decrease of CDC42 activity and the demyelination of peripheral nerves ultimately. However, in this study, mir-23a expression and function were only studied by using patients’ peripheral blood and cell lines cultured in vitro. The expression and function of mir-23a in vivo and patients’ ovarian cells still need further verification.

In addition, recent studies have shown that FGD4 expression in prostate cancer clinical samples is significantly up-regulated compared with the normal group, and down-regulation expression of FGD4 in prostate cancer cell lines can cause cell cycle arrest and proliferation reduction [28]. It seems that FGD4 is also involved in the tumorigenesis of nasopharyngeal carcinoma due to its activation of CDC42 [29]. Studies have shown that activated CDC42 regulates downstream signals such as PAK-1, WASP and ACK. PAK-1, as a serine/threonine kinase, was originally identified as a protein that interacts with CDC42 and was subsequently found to serve as a downstream node for various oncogenic signaling pathways. Studies have shown that the CDC42/PAK-1 signaling pathway involved in cell cycle, proliferation and apoptosis regulation [30]. Our study found that miR-23a affects the expression of FGD4 as well as the protein levels of activated CDC42 (GTP bround) and p-PAK-1. Therefore, we hypothesized that miR-23 regulated CDC42/PAK-1 signaling pathway by targeting FGD4 expression, ultimately affecting apoptosis of cov434 cells.

In conclusion, our study reveals that the serum level of miR-23a is significantly down-regulated in PCOS patients, and that miR-23a participates in the regulation of proliferation and apoptosis of cov434 cells through target FGD4, which may have potential for clinical treatment of PCOS patients.

## Data Availability

All data generated or analysed during this study are included in this published article.
